# Erianin promotes endogenous neurogenesis in traumatic brain injury rats

**DOI:** 10.1038/s41598-023-50573-8

**Published:** 2024-02-19

**Authors:** Qingquan Li, Xiaokui Gan, Ming Zhang, Guangmin Zhang, Yingbin Li, Liang Gao

**Affiliations:** 1 Department of Neurosurgery, Second Affiliated Hospital of Nanjing Medical University, Nanjing, China; 2 Department of Shanghai Tenth People’s Hospital Clinical Medical College, Nanjing Medical University, Nanjing, China; 3 Department of Neurosurgery, Shanghai Tenth People’s Hospital, Tongji University, No. 301 Extend Middle Road, Shanghai, 200072 China

**Keywords:** Cellular neuroscience, Neurogenesis, Regeneration and repair in the nervous system

## Abstract

The objective of this study was to explore the positive influence and potential mechanism of Erianin on the recovery of brain cells following a traumatic brain injury (TBI). TBI rat models were prepared and treated with Erianin injection via tail vein. The assessment included evaluating the rats' levels of oxidative stress, inflammation, neuronal damage, mitochondrial damage, neuronal regeneration, transformation of pro-inflammatory microglial cells, activation status of the ERK signal pathway, and the functionality of their learning and memory. After administering Erianin, there was a suppression of oxidative stress, inflammation, nerve cell damage, and mitochondrial damage in the TBI rats. Additionally, there was an increase in neuronal regeneration in the cortex and hippocampus, inhibition of pro-inflammatory microglial cell transformation in the cortex, improvement in learning and memory function in TBI rats, and simultaneous inhibition of the activation of the ERK1/c-Jun signal pathway. The findings suggest that Erianin has the potential to reduce oxidative stress and inflammatory reaction in rats with TBI, safeguard nerve cells against apoptosis, stimulate the growth of new neural cells, ultimately enhancing the cognitive abilities and memory function of the rats. The inhibition of the ERK signaling pathway could be closely associated with these effects.

## Introduction

External mechanical forces cause traumatic brain injury (TBI), which refers to an injury to the brain. Based on the pathological process, TBI can be divided into two stages: primary brain injury and secondary brain injury^1^. Secondary brain injuries typically occur several minutes or days after primary brain injury, involving a complex series of cellular and biochemical processes. These include oxygen free radical overload, release of excitatory amino acids, massive release of inflammatory factors, breakdown of the blood–brain barrier, overload of calcium and sodium ions, and mitochondrial dysfunction. As a result, disordered arrangement, apoptosis, and loss of nerve cells can occur^2,3^. Despite extensive research on the development of TBI, current pharmaceutical interventions to safeguard cells and prevent their demise remain inadequate. Consequently, a significant proportion of individuals with TBI experience enduring disabilities or cognitive deficits, imposing substantial economic hardships on both society and families.

Erianin mainly originates from Dendrobium chrysotoxum and Dendrobium nobile, belonging to the class of benzene compounds and having a wide range of pharmacological effects^4,5^. Various biological processes have been discovered to involve its participation, including programmed cell death, angiogenesis, and antioxidant characteristics. It serves a specific function in the investigation of diseases such as tumors, inflammation, diabetic nephropathy, retinal diseases, and ulcerative colitis. It is anticipated to emerge as a promising medicinal remedy for various ailments^5–10^. By inhibiting the signaling transduction of Toll-like receptor (TLR) 4 and the transduction of transcription activation factor 3 (STAT3), Erianin can regulate the levels of inflammation, oxidative stress-related factors, and immune chemoattractant factors in serum and colon tissue. This helps alleviate cell peroxidation damage, immune inflammatory response, and relieve symptoms of ulcerative colitis^7^. Erianin has the ability to relieve diabetic retinopathy by blocking the ERK1/2-NF-κB signaling pathway triggered by elevated blood glucose levels, thus reducing retinal inflammation caused by the activation of microglia^8^. By preventing the activation of IL-6 and IL-8, Erianin has the potential to decrease oxidative harm caused by HG in renal tubular epithelial cells, potentially functioning as an antioxidant^6^. According to the analysis results from the Comparative TOXicogenomics Database (CTD), it appears that Erianin could potentially have a significant impact on TBI^11^. However, the exact impact of Erianin on central nervous system damage remains uncertain. The objective of this study was to investigate the enhancing impact and potential mechanism of Erianin on the generation of new nerve cells within the brain after TBI. The findings aim to establish a scientific and practical foundation for the use of Erianin as a treatment for TBI.

## Results

### Erianin inhibited oxidative stress levels after TBI

**Figure 1:**
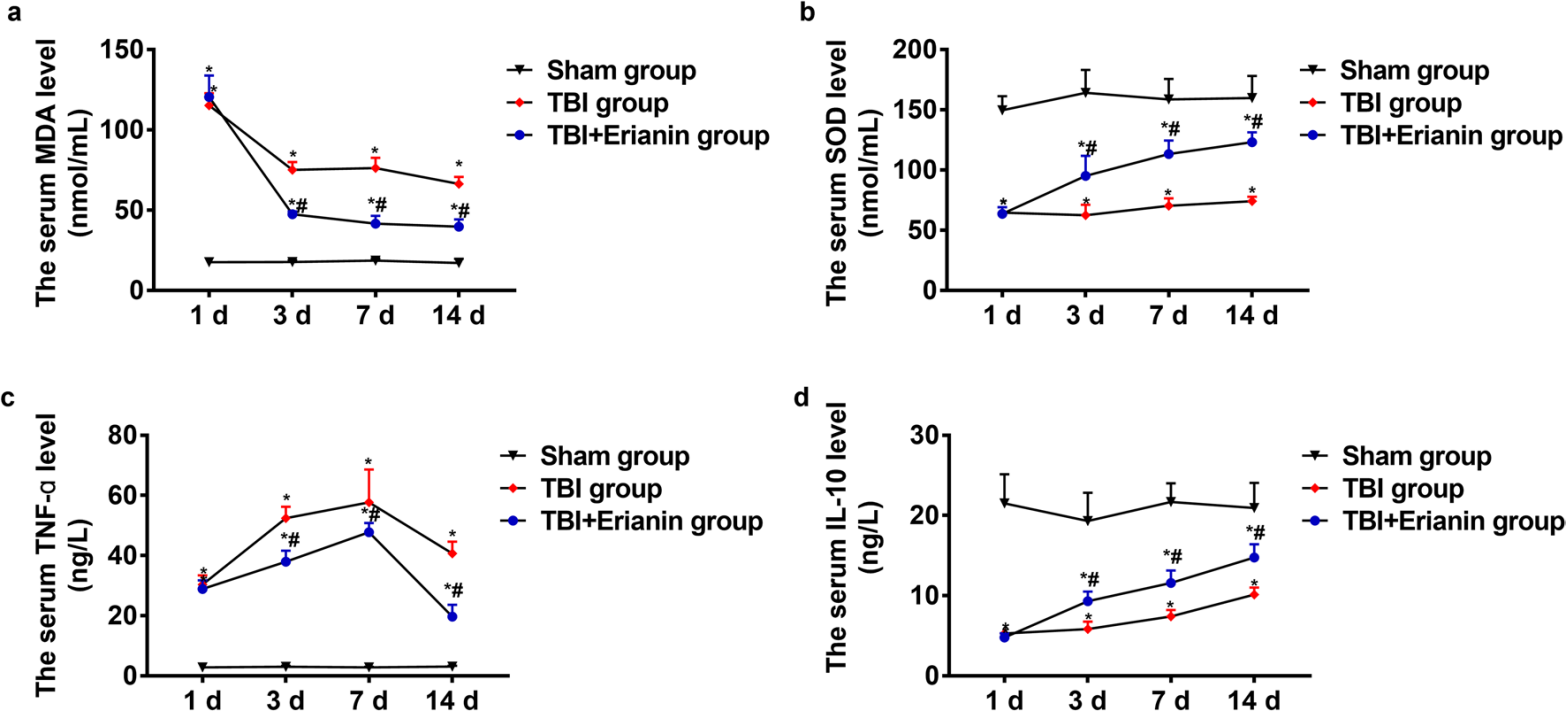
Erianin inhibited oxidative stress and inflammation levels after TBI. (**a**) MDA levels in three different groups. (**b**) The level of SOD in three groups. (**c**) The levels of TNF-α in the serum were measured in three different groups. (**d**) The serum level of IL-10 in three groups. * vs. Sham group, *P* < 0.05; # vs. TBI group* P* < 0.05.

At the time points of 1, 3, 7, and 14 days after TBI, there were no significant changes in serum levels of MDA and SOD in the Sham group (P > 0.05). On the first day after injury, the TBI group and the TBI + Erianin group showed elevated levels of MDA in the serum. The MDA levels in the TBI group decreased on the third, seventh, and fourteenth day (P < 0.05), with no significant difference on the third and seventh day (P > 0.05). The MDA levels in the TBI + Erianin group gradually decreased on the third, seventh, and fourteenth day (P < 0.05), showing a more significant decrease than the TBI group (P < 0.05), but it did not reach the level of the Sham group (P < 0.05) (Fig. 1a). On the first day after injury, the serum levels of SOD in the TBI group and the TBI + Erianin group decreased. The SOD levels in the TBI group remained at a lower level on the third day and gradually increased on the 7th and 14th day (P < 0.05). The SOD levels in the TBI + Erianin group gradually increased on the third, seventh, and fourteenth day, showing a more significant increase than the TBI group (P < 0.05), but it did not reach the level of the Sham group (P < 0.05) (Fig. 1b).

### Erianin inhibited inflammation levels after TBI

At the time points of 1, 3, 7, and 14 days after TBI, there were no significant changes in serum levels of TNF-α and IL-10 in the Sham group (P > 0.05). On the first day after injury, the TBI group and the TBI + Erianin group showed elevated levels of TNF-α in the serum. The levels of TNF-α in the TBI group and the TBI + Erianin group continued to rise on the third and seventh days, but the TBI + Erianin group had relatively lower levels. On the fourteenth day, the levels of TNF-α in the TBI group and the TBI + Erianin group decreased, with a more significant decrease in the TBI + Erianin group, but they did not reach the levels of the Sham group (P < 0.05) (Fig. 1c). On the first day after injury, the serum levels of IL-10 in the TBI group and the TBI + Erianin group significantly decreased (P < 0.05), gradually increasing on the third, seventh, and fourteenth days, with the TBI + Erianin group showing a more significant increase compared to the TBI group (P < 0.05), but they did not reach the levels of the Sham group (P < 0.05) (Fig. 1d).

### Erianin reduced the degree of TBI nerve damage

**Figure 2:**
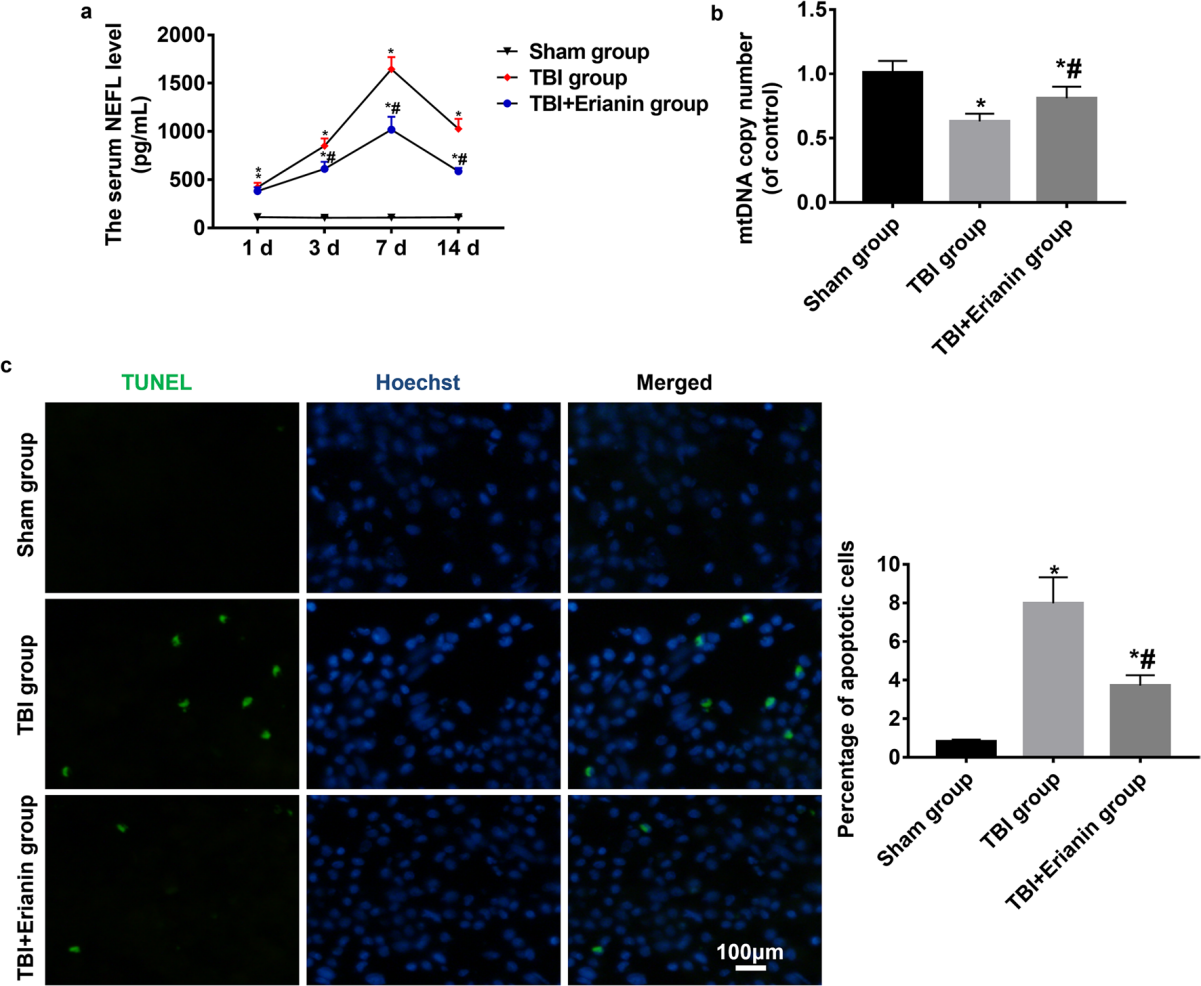
Erianin reduced the degree of TBI nerve damage. (**a**) The levels of NEFL in serum were measured in three different groups. (**b**) The number of mtDNA copies in three different groups. (**c**) The percentage of cells undergoing apoptosis in three different groups. * vs. Sham group* P* < 0.05; # vs. TBI group* P* < 0.05. Bar = 100 μm.

At the relevant time points of testing, the serum NEFL levels in the Sham group did not show significant changes (P > 0.05). On the first day after TBI, the NEFL levels in the blood of the TBI group and TBI + Erianin group increased, and continued to increase on the third and seventh days, but the NEFL levels in the TBI + Erianin group were relatively lower. The NEFL levels in the blood of the TBI group and TBI + Erianin group decreased on the fourteenth day (P < 0.05), with a more significant decrease in the TBI + Erianin group, but did not reach the level of the Sham group (P < 0.05) (Fig. 2a). The mtDNA copy number of the cortex surrounding the injury in the TBI group showed a significant decrease (*P* < 0.05) compared to the Sham group. Following administration of Erianin, the mtDNA replication count in the cortex adjacent to the injury significantly rose (*P* < 0.05), yet it remained below the level observed in the Sham group (*P* < 0.05) (Fig. 2b). The percentage of apoptotic cells in the cortex surrounding the injury significantly increased in the TBI group compared to the Sham group (*P* < 0.05). Following the administration of Erianin, there was a notable reduction (*P* < 0.05) in the proportion of apoptotic cells in the cortex adjacent to the injury site in the TBI + Erianin group; however, it remained higher level than that observed in the Sham group (Fig. 2c).

### Erianin inhibited the transformation of microglia cells into proinflammatory types after TBI

**Figure 3:**
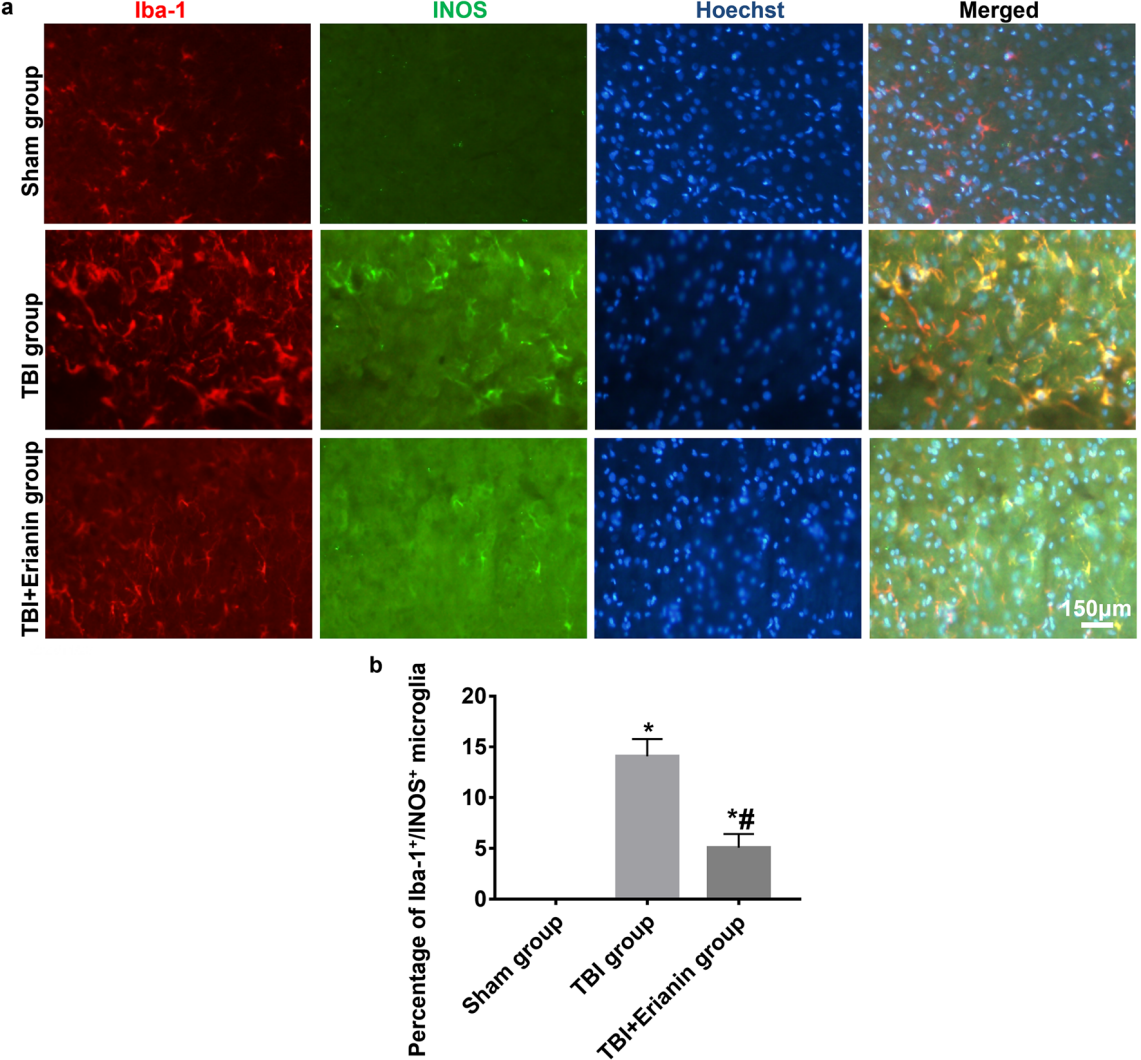
After TBI, Erianin suppressed the conversion of microglia cells into pro-inflammatory phenotype. (**a**) The Iba-1/INOS immunofluorescence double labeling microglia cells in three groups. (**b**) The percentage of positive microglia cells with Iba-1/INOS double labeling among the three groups. * vs. Sham group* P* < 0.05; # vs. TBI group* P* < 0.05. Bar = 150 μm.

No cortex showed the presence of positive microglia cells with double labeling of Iba-1/INOS in the Sham group. A significant quantity of microglia cells with dual labeling of Iba-1/INOS were observed in the cortex surrounding the injury site in rats with TBI from the TBI group. Following the administration of Erianin, there was a notable reduction in the number of microglia cells expressing both Iba-1 and INOS in the cortical region surrounding the site of injury in the TBI + Erianin group (Fig. 3a). When comparing the three groups pairwise, there were notable variations in the proportion of microglia cells labeled with Iba-1/INOS that tested positive (*P* < 0.05) (Fig. 3b).

### Erianin promoted endogenous neural regeneration after TBI

**Figure 4:**
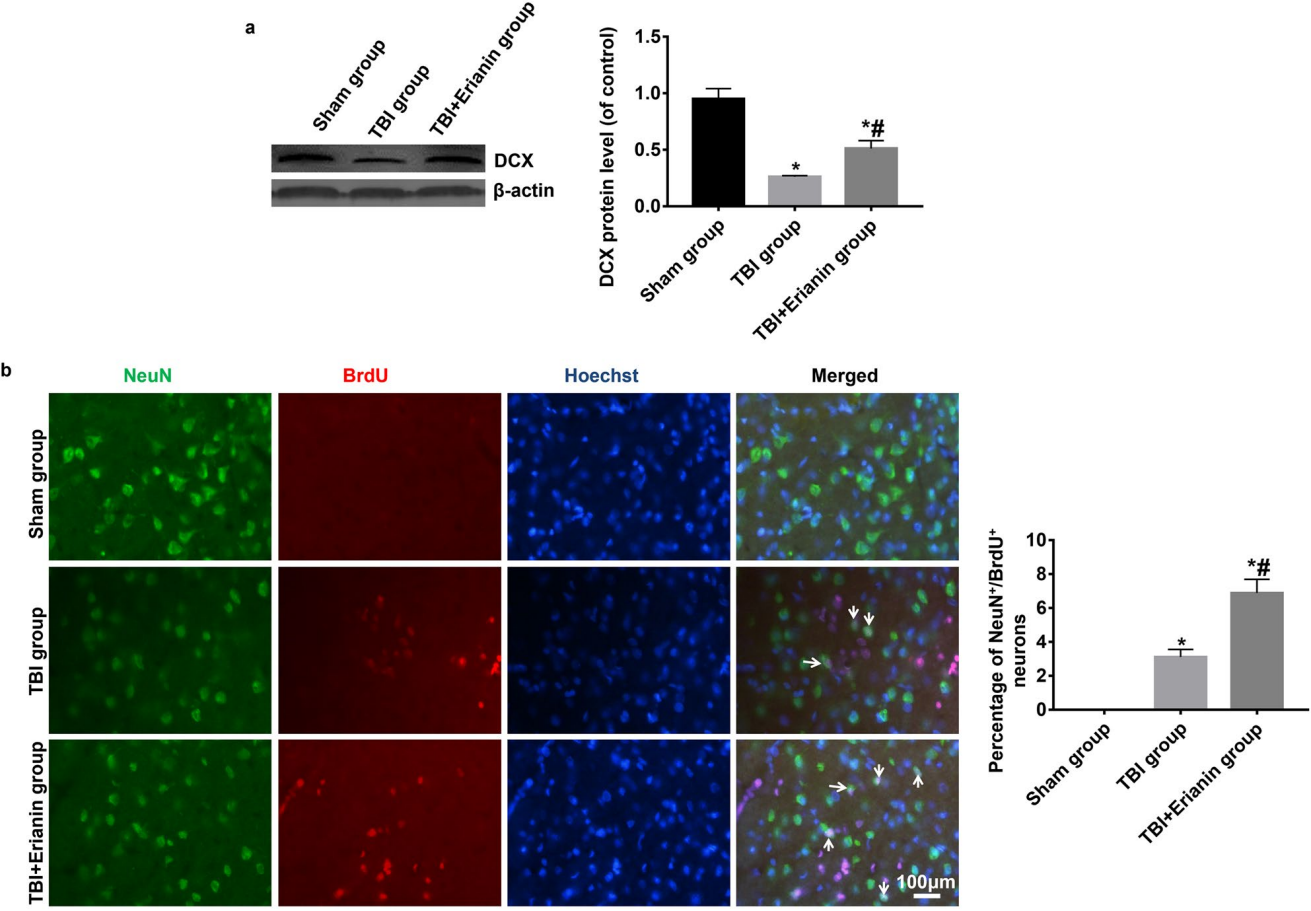
Erianin promoted endogenous neural regeneration after TBI. (**a**) The DCX protein level in hippocamp. (**b**) The percentage of positive neurons with NeuN/BrdU double labeling among the three groups. * vs. Sham group* P* < 0.05; # vs. TBI group* P* < 0.05. Bar = 100 μm.

The western blot results indicated a decrease in the level of the DCX protein in the injured side hippocampus of the TBI group compared to the Sham group (*P* < 0.05). However, the DCX protein level in the injured side hippocampus showed improvement following Erianin treatment in the TBI + Erianin group, although it did not reach the level of the Sham group (*P* < 0.05) (Fig. 4a). No cortex neurons with double labeling of NeuN/BrdU were observed in the Sham group. A small number of neurons that were positive for both NeuN and BrdU labeling were observed in the cortex near the injury site in rats with TBI from the TBI group. The administration of Erianin resulted in a significant increase in the number of neurons labeled with NeuN/BrdU in the cortical region surrounding the site of injury in the TBI + Erianin group. When comparing pairwise, there were notable variations in the proportion of neurons labeled with NeuN/BrdU among the three groups (Fig. 4b).

### Erianin inhibited the ERK1/c-Jun signal pathway activation

**Figure 5:**
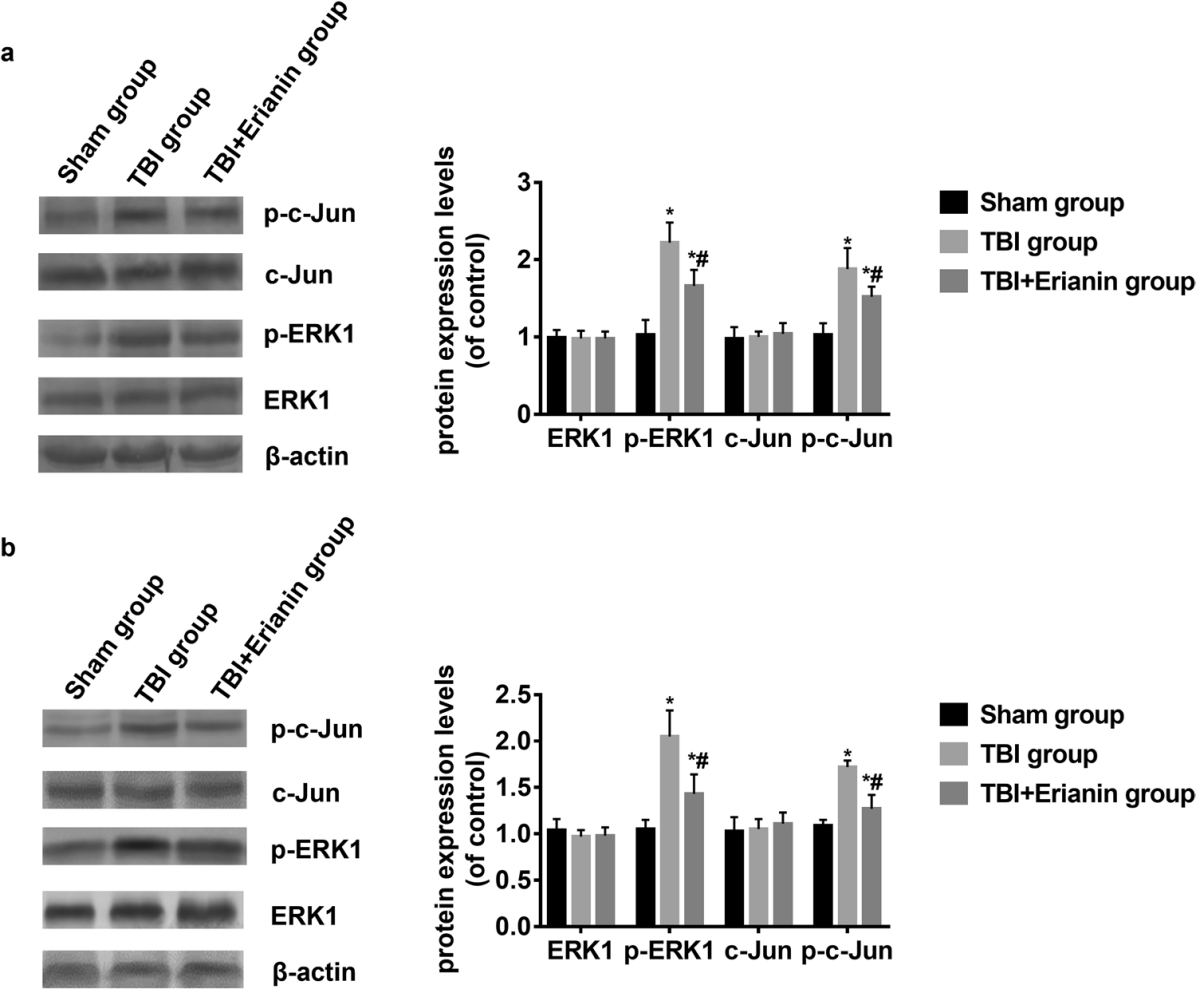
Erianin inhibited the ERK1/c-Jun signal pathway activation. (**a**)The levels of ERK1 protein, C-Jun protein, p-ERK1 protein, and p–c-Jun protein in hippocamp were compared among the three groups. (**b**) The levels of ERK1 protein, C-Jun protein, p-ERK1 protein, and p–c-Jun protein in the cortex were compared among the three groups. * vs. Sham group* P* < 0.05; # vs. TBI group* P* < 0.05.

No significant variation in the protein levels of ERK1 and C-Jun in hippocampus was observed among three groups, as indicated by the Western blot findings. In the TBI group, the levels of p-ERK1 and p–c-Jun proteins in the injured side hippocampus increased compared to the Sham group (*P* < 0.05). However, in the TBI + Erianin group, the levels of p-ERK1 and p–c-Jun proteins in the injured side hippocampus recovered after Erianin treatment, but they still did not reach the level of the Sham group (*P* < 0.05) (Fig. 5a). Figure 5b illustrates similar findings in the cortex as well.

### Erianin improved the learning and memory function of rats after TBI

**Figure 6:**
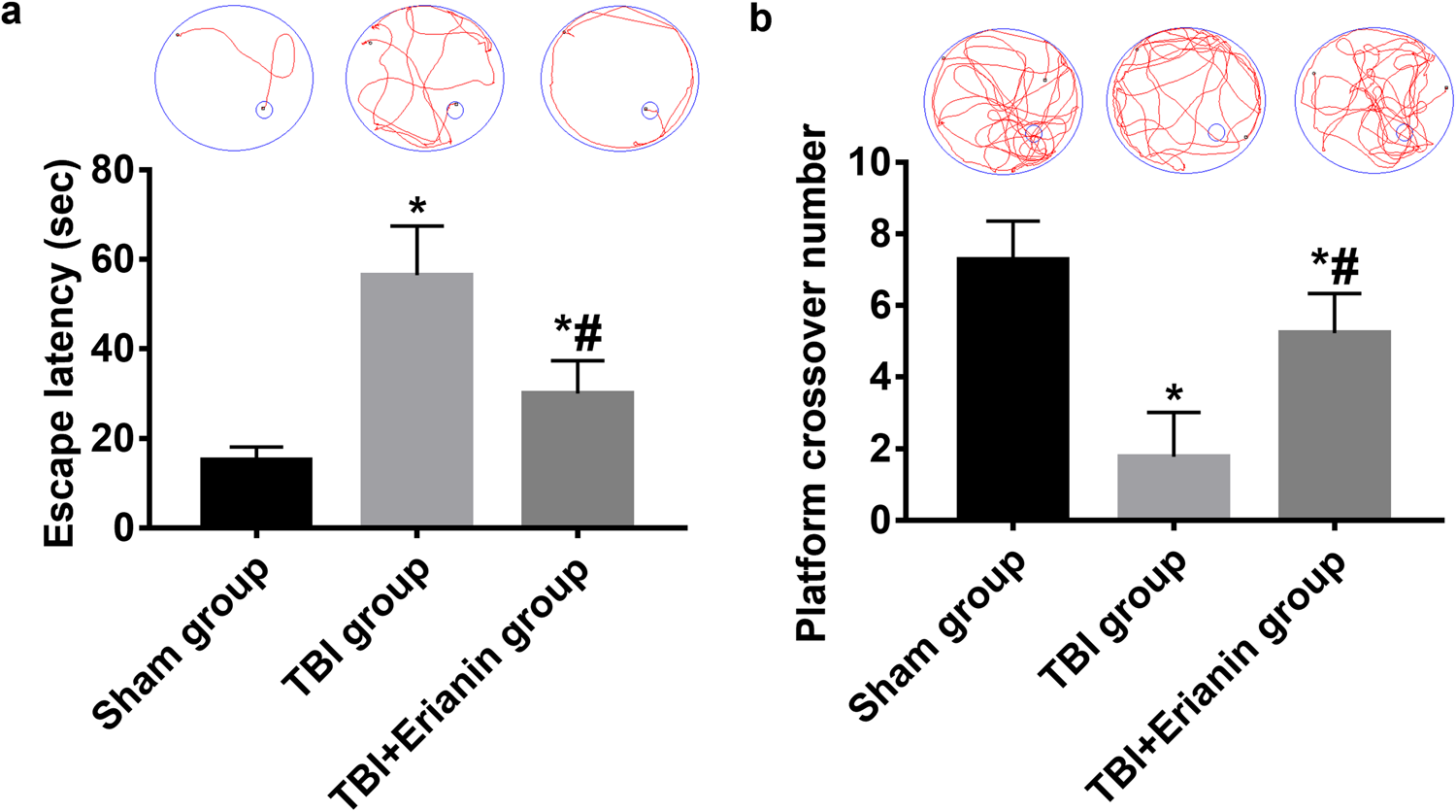
Erianin enhanced the cognitive abilities of rats following TBI. (**a**) The rats' escape latency in the navigation experiment. (**b**) The number of rats crossing over platforms in space exploration experiments. * vs. Sham group* P* < 0.05; # vs. TBI group* P* < 0.05.

After experiencing TBI, rats showed enhanced learning and memory abilities with the administration of Erianin. In the navigation experiment, it was observed that the TBI rats in the TBI group had a longer escape latency compared to the Sham group (*P* < 0.05). However, the TBI rats in the TBI + Erianin group had a shorter escape latency than the TBI group, although it was still longer than the Sham group (*P* < 0.05) (Fig. 6a). In the space exploration experiment, the findings revealed that the Sham group had the highest number of rats crossing the platform compared to the other two groups (*P* < 0.05). On the other hand, the TBI group had the lowest number of platform crossovers (*P* < 0.05). Interestingly, the TBI rats in the TBI + Erianin group showed a slight increase in platform crossovers after receiving Erianin treatment, although it remained lower than the Sham group (*P* < 0.05) (Fig. 6b).

## Discussion

TBI is a damage to the nervous system caused by mechanical deformation of the skull, brain tissue, and blood vessels after the head receives external force, causing temporary or permanent functional disorders. Common causes of injury include traffic accidents, falls, and acts of violence^12,13^. At present, the primary focus in treating TBI is to avert initial harm and halt subsequent damage. Although extensive research has been conducted regarding the inhibition of neuroinflammatory response, correcting iron overload, reducing oxidative stress, and restoring the blood–brain barrier, there is still no specific and effective drug or method to restore and repair damaged neural function. Erianin, a low molecular weight biphenyl compound, exhibits a wide range of pharmacological effects, making it a potential therapeutic drug for various diseases^5–10^.

The study demonstrated that the administration of Erianin effectively reduces the serum concentrations of MDA and TNF-α in rats with TBI, while simultaneously increasing the levels of SOD and IL-10 in the serum. These findings indicate that Erianin has the potential to alleviate oxidative stress and inflammation following TBI. Additionally, our findings revealed that Erianin treatment suppresses the serum concentrations of NEFL, which serve as biomarkers for nerve cell damage in the blood of rats with TBI. Moreover, through TUNEL analysis, we observed a significant decrease in the proportion of apoptotic cells in the cortex surrounding the injury following Erianin treatment. The above-mentioned results indicate the Erianin has the potential to protect nerve cells from injury. Mitochondria play a crucial role in supplying cells with energy in the form of adenosine triphosphate (ATP), and they have a significant impact on various cellular processes. Reduced mitochondrial content is often associated with impaired mitochondrial function. Impaired mitochondrial function is a significant factor contributing to neuronal death after secondary brain injury^14^. After TBI, brain tissue undergoes ischemia and hypoxia, leading to a cerebral energy crisis. This, in turn, leads to an increase Oxidative phosphorylation (OXPHOS) levels, resulting in the release of large amount of reactive oxygen species (ROS) from mitochondria, further exacerbating mitochondrial dysfunction^15^. The mtDNA copy number in the cortex surrounding the injury was evaluated using qPCR in this investigation. The qPCR findings indicated a significant increase in the mtDNA copy number in the cortex surrounding the injury following Erianin treatment. This result indicate that Erianin can protect the mitochondria from injury in the TBI.

As a long-standing global public health challenge, TBI have serious consequences. Approximately one-third of TBI patients die, while another one-third experience severe disabilities or remain in a vegetative state. Even individuals who have survived or are dealing with mild TBI may encounter various levels of headaches, depression, as well as behavioral, cognitive, and emotional functional disorders. Additionally, there is a significant likelihood and increased occurrence of neurodegenerative disorders such as Alzheimer's, Parkinson's, and epilepsy^16–19^. The learning and memory function of TBI rats was assessed using the MWM test in this research. The findings indicated a significant decline in the cognitive and mnemonic capacity of TBI rats. However, following administration of Erianin, there was a notable enhancement in their cognitive and mnemonic capabilities. The findings indicate that Erianin has the potential to enhance the restoration of cognitive abilities and memory function in rats with TBI.

Neuroinflammation is a defining feature of TBI, in which resident glial cells (microglia and astrocytes) become activated, immune cells are recruited, and inflammatory mediators are released within the brain. Microglia, as the initial defense mechanism in neuroinflammation, promptly respond to damage-related molecular patterns released following brain trauma and relocate to the site of injury through an ATP-dependent process. Early activated microglia are believed to have neuroprotective effects and can clear cellular debris. However, in the chronic phase of TBI, many microglia remain in an activated state, which is often considered harmful^20,21^. Microglia, immune cells found in the brain, exhibit a wide range of intricate effects that vary from neurotrophic to neurotoxic, depending on their state of activation. Typically, microglia are categorized into proinflammatory M1 type and anti-inflammatory M2 type based on the classification of macrophages^22^. Toll-like receptor and γ-interferon signaling pathways typically induce M1 activation, which is characterized by a pro-inflammatory and neurotoxic condition.M1 microglia have the ability to generate pro-inflammatory cytokines like TNF-α, IL-6, and IL-1β, as well as chemokines. Additionally, they exhibit the presence of NADPH oxidase and matrix metalloproteinase 12^23^. Activation of microglia in M2 state exhibits anti-inflammatory and reparative properties. M2 microglia possess the ability to stimulate the production of arginase 1, release growth factors, and facilitate the secretion of anti-inflammatory cytokines like IL-10 and TGF-β^23^. Nevertheless, there is still a debate surrounding M1 and M2 microglia, as evidence suggests that the polarization of glial cells is complex and involves significant overlap in gene expression rather than following a simplistic linear spectrum^24^. For this investigation, we utilized Iba-1/INOS immunofluorescence to identify the activation of microglia. The findings indicated the presence of activated pro-inflammatory microglia in the cortex surrounding the injured regio, even after 20 days post-TBI. Following the administration of Erianin, there was a notable reduction in the number of microglia cells displaying positive Iba-1/INOS labeling in the cortex surrounding the injury location. This suggests that Erianin has the ability to hinder the conversion of microglia cells into pro-inflammatory phenotypes subsequent to TBI.

For many years, people have often been misled by a well-known belief that no new neurons form in the brains of mammals after they are born ^25^, mainly because of the limitations in research technology and other cfactors. However, in 1962, Altman ^26^ actually utilized thymidine-H(3) injection to label recently formed cells in the rat brain affected by trauma, revealing a small number of identified newborn neurons and neural precursor cells in the damaged region. However, this discovery did not gain significant recognition within the academic community at the time. Over the past few years, there have been continuous reports of the presence of native neurons in the cortical area of TBI affected region, and their occurrence rate can be greatly enhanced by appropriate intervention of relevant factors^27–30^. During this investigation, it was observed that following TBI, a small number of neurons displaying positive NeuN/BrdU double labeling emerged in the cortical region surrounding the site of injury. Following the administration of Erianin, there was a notable rise in the number of neurons displaying positive NeuN/BrdU double labeling. The findings validate that Erianin has the ability to enhance the natural formation of new neurons in the cerebral cortex following TBI. Research has consistently shown that adult rodents in a physiological state possess neural stem cells/neuroblasts in a dormant or active state within the subventricular zone (SVZ) and sub-granular zone (SGZ) of the dentate gyrus^31,32^. By employing a unique detection technique, Spalding et al.^33^ discovered that the genomic DNA of the human hippocampus shows a 1.75% annual renewal rate of the carbon-14 isotope. Furthermore, the pace of renewal slightly declines with age. Patients with brain injuries tend to have an increase in hippocampus neurogenesis ^34^. However, research on rodent TBI models has produced contradictory results. Some findings have discovered a notable rise in the extent of innate neurogenesis within the hippocampus following TBI^35–37^, whereas others have noted a substantial decline^35,38,39^. The contradictory results may be related to differences in the TBI models, degree of injury and the time point of observation. In this study, we found that the level of DCX protein in the injured side of the hippocampus in rats significantly decreased 20 days after TBI, but increased after treatment with Erianin. This result suggests that Erianin can promote neurogenesis in the hippocampus after TBI.

ERK1 and ERK2, known as extracellular regulated protein kinases, play a vital role in transmitting signals from receptors on the cell surface to the nucleus. Once phosphorylated and activated, ERK1/2 move from the cytoplasm to the nucleus. In the nucleus, they are involved in the transcriptional activation of Elk-1, ATF, Ap-1, c-fos, and c-Jun, and contribute to different biological responses^40–43^. Multiple researches have showed that following a TBI, the ERK signal becomes excessively activated, playing a role in the biological mechanism of secondary brain damage resulting from TBI^44,45^. For this investigation, Western blot was employed to examine the protein quantities of ERK1, p-ERK1, C-Jun, and p–c-Jun following TBI. The findings indicate that TBI did not induce significant alterations in the protein levels of ERK and c-Jun in the hippocampus and cortex. Nevertheless, there was a notable rise in the concentrations of p-ERK and p–c-Jun. Subsequent administration of Erianin resulted in a significant decline in the levels of p-ERK and p–c-Jun. This result suggests that Erianin can inhibit the ERK1/c-Jun signal pathway activation after TBI. However, the regulatory role of this signaling pathway needs further clarification through subsequent in vitro cell research.

To summarize, Erianin has the capability to reduce oxidative stress and inflammatory reaction in rats with TBI, safeguard nerve cells against apoptosis, stimulate the growth of new neural cells, ultimately enhancing the cognitive abilities and memory function of rats. The inhibition of the ERK/c-Jun signaling pathway could be closely associated with these effects.

## Materials and methods

### Rats and groups

A total of 54 SD rats, weighing between 220 and 250 g and of male, were acquired from the Animal Experiment Center at Nanjing Medical University. All experiments and methods were performed in accordance with the Animal Research: Reporting of In Vivo Experiments (ARRIVE) guidelines^46^. We declared that all methods were carried out in accordance with the relevant guidelines and regulations. The rats were divided into three groups, namely the TBI + Erianin group, TBI group, and Sham group, each consisting of 18 rats. The TBI + Erianin group and solvent group were used to prepare TBI models. To prepare the cranial fluid pressure injury model in accordance with Yi et al.'s study^27^, the American AmScien Instruments Company FP302 controlled device was used. The mice were heavily sedated using isoflurane gas and their skulls were fastened to a stereotactic device. Following the removal of hair and sterilization of the head, a 5 mm diameter circular bone opening was created 3.5 mm in front of the bregma and 3.0 mm to the left of the sagittal suture, while keeping the dura mater undisturbed. The SD rats were given a peak shock pressure of 1.5 atm (1 atm = 101.3 kPa) to induce a cranial injury on the left side. In contrast, the Sham group only had a bone window opened without creating a brain injury model. All rats were administered with BrdU (50 mg/kg, Bid) via intraperitoneal injection to label newborn neurons, continuously for 5 days after surgery. Rats of TBI + Erianin group were intravenously injected with Erianin (Sichuan Victory Biotechnology Co. Ltd, China, 2 mg/kg/day, dissolved in DMSO) for 14 day, and rats in the other two groups were intravenously injected with equal DMSO. Drug dosage selection was based on previous study^47^. The rats were given unlimited food and water and were kept at a temperature of 22 ± 2 °C, with a relative humidity of 50 ± 5%, and a 12-h light/dark cycle.

### Serum index detection

At 1, 3, 7, and 14 days post-injury, blood samples were gathered from the tail vein of every rat, with a volume of 1 ml. Serum malondialdehyde (MDA) and superoxide dismutase (SOD) levels were assessed using MDA and SOD kits (Beyotime Biotechnology, China) in accordance with the manufacturer's guidelines. ELISA kits (Abcam, UK) were used to measure the levels of serum neurofilament light (NEFL), TNF-α, and IL-10, following the manufacturer's instructions.

### Morris water maze (MWM) tests

The cognitive functions of rats were assessed using the MWM equipment provided by Shanghai Yanjiang BioTech Co., Ltd., China. The navigation experiment commenced 15th day after the TBI to monitor the escape latency over a period of 4 days. A space exploration experiment was carried out on the 19th day following the TBI to record the number of platform crossovers. The data underwent automatic analysis using the Animal Behavior Video Analysis System provided by Shanghai Yanjiang BioTech Co., Ltd., a company based in China.

### qPCR

DNA was extracted from the cortical tissues of rats after MWM tests, following the guidelines provided by the DNA extraction kit (Beyotime Biotechnology, China). PCR products were amplified in a 20 μl reaction containing 2 SYBR Mix (10 μl) and 2 μl of each primer, RNase-Free ddH_2_O 6 μl, using 25 ng of DNA. In order to evaluate the mtDNA replication level, the NADH dehydrogenase 1 (ND1) gene was amplified, while the nuclear-encoded apolipoprotein B (APOB) gene was employed for the purpose of normalization. The primer sequences are as follows: ND1 sense 5′-TGAATCCGAGCATCCTACC-3′, anti-sense 5′-ATTCCTGCTAGGAAAATTGG-3′; APOB sense 5′-CGTGGGCTC CAGCATTCT A-3′, antisense 5′-TCACCAGTC ATT TCT GCC TTT G-3′. To calculate the mtDNA copy number, the 2^−ΔΔCt^ analysis technique was employed^48^.

### Western blot

The RIPA Lysis Buffer (Beyotime Biotechnology, China) was utilized for extracting total protein from fresh cortical tissues surrounding the injured cortex or injured side hippocampal tissue of rats 20 days after injury. The protein concentration was measured using the BCA protein quantification kit (Beyotime Biotechnology, China). The protein samples were combined with loading buffer, subjected to heating at a temperature of 95 °C for a duration of 10 min in a water bath, and subsequently transferred to ice for a period of 10 min prior to storage at a temperature of −20 °C. The protein samples were separated using gel electrophoresis with SDS-PAGE, then transferred to a PVDF membrane. After being blocked with 5% skim milk in TBST, the blots were cut prior to hybridisation with antibodies. Then the samples were incubated overnight at 4 °C with the primary antibody, followed by a 1.5-h incubation at room temperature with the secondary antibody. The primary antibodies used were rabbit anti-DCX (1:500, Abcam, UK), rabbit anti-extracellular regulated protein kinases 1 (ERK1) (1:600, Abcam, UK), rabbit anti-p-ERK1 (1:600, Abcam, UK), rabbit anti-c-Jun (1:800, Abcam, UK), rabbit anti-p–c-Jun (1:800, Abcam, UK), and mouse anti-β-actin (1:800, Abcam, UK). The secondary antibody used was IRDye 700-conjugated affinity-purified goat anti-mouse (1:4000, Rockland Immunochemicals, USA), and IRDye 800-conjugated affinity-purified goat anti-rabbit (1:4000, Rockland Immunochemicals, USA) was also used. The expression level of the protein was analyzed using the Odyssey laser scanning system from LICOR Inc., USA.

### TUNEL

Following a 20-day period after the injury, certain rats underwent formaldehyde perfusion and their brains were frozen to facilitate coronal sectioning. These sections were then utilized for TUNEL and immunofluorescence examinations. The TUNEL assay kit (Beyotime Biotechnology, China) was employed to identify the apoptosis of nerve cells in the cortical region adjacent to the injury, following the guidelines provided by the manufacturer. To counterstain cell nuclei, Hoechst 33342 (1:2000, ThermoFisher Scientific, Inc.) was applied for 0.5 h at room temperature, ensuring protection from light. Afterwards, the fluorescence microscope (Leica DMR, German) was used to observe the positive cells.

### Immunofluorescence

Using prepared brain tissue slices for immunofluorescent staining. The brain slice was obstructed using a blocking buffer (Beyotime Biotechnology, China), supplemented with primary antibodies, and left to incubate overnight at 4 ℃. Subsequently, secondary antibodies were introduced and incubated for 6 h at room temperature in a darkroom. The main antibodies employed were mouse anti-Iba-1 (at a dilution of 1:800, bought from Abcam in the United Kingdom), rabbit anti-INOS (at a dilution of 1:400, also from Abcam in the United Kingdom), rabbit anti-NeuN (at a dilution of 1:400, from Abcam), and mouse anti-BrdU (at a dilution of 1:200, bought from Abcam).The secondary antibodies used were goat anti-rabbit conjugated with Alexa Fluor^®^ 488 (at a dilution of 1:1000, obtained from Abcam) and goat anti-mouse conjugated with Alexa Fluor^®^ 568 (at a dilution of 1:800, obtained from Abcam).Additionally, Hoechst 33342 (at 1:2000, from ThermoFisher Scientific, Inc.) was employed to counterstain cell nuclei for half an hour at room temperature in a light-protected environment. Using a fluorescence microscope (Leica DMR, from Germany), positive cells were observed.

### Statistical analysis

The study utilized the SPSS 21.0 software to conduct data analysis, and the results were presented as the mean ± standard deviation. To compare the groups, the study used one-way analysis of variance (ANOVA) followed by Tukey's test. GraphPad Prism 7 software was employed to generate statistical graphs. A significance level of P < 0.05 was chosen for statistical calculations (Supplementary Information).

### Ethics approval

The study was approved by the Medical Ethics Committee of the Second Affiliated Hospital of Nanjing Medical University.

### Supplementary Information

Supplementary Information 1.

Supplementary Information 2.

## Data Availability

The datasets used and/or analyzed during the current study are available from the corresponding author on reasonable request.
