# Supplementary material for: Erianin promotes endogenous neurogenesis in traumatic brain injury rats
Source: Sci Rep. 2024 Feb 19;14:4108. doi: 10.1038/s41598-023-50573-8 (PMC10876537; doi:10.1038/s41598-023-50573-8)
Supplement: Supplementary file 2 — Supplementary Information 2. [file 41598_2023_50573_MOESM2_ESM.pdf]

Figure-4a Primary WB bands

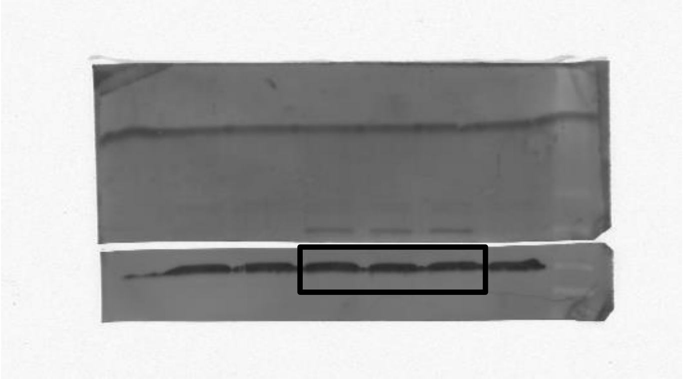

β-actin 42 kDa

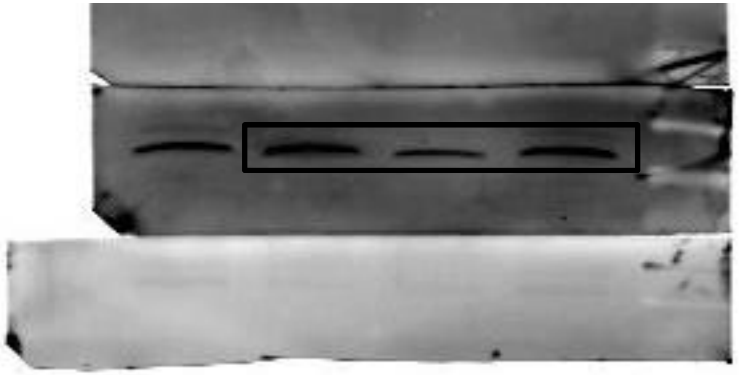

DCX 26 kDa

Figure-5a Primary WB bands

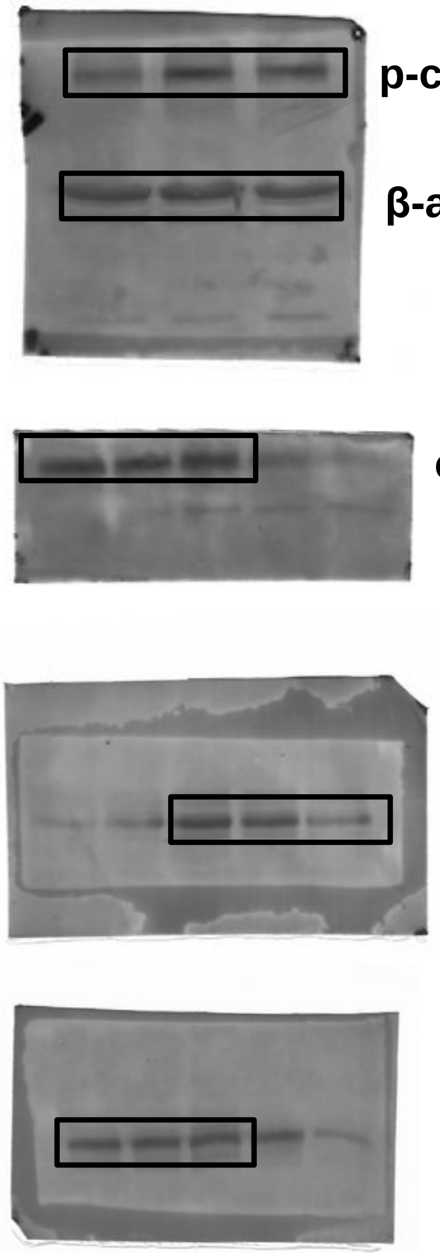

p-c-Jun 50 kDa

β-actin 42 kDa

c-Jun 39 kDa

p-ERK1 44 kDa

ERK1 44 kDa

Figure-5b Primary WB bands

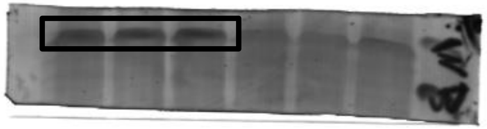

β-actin 42 kDa

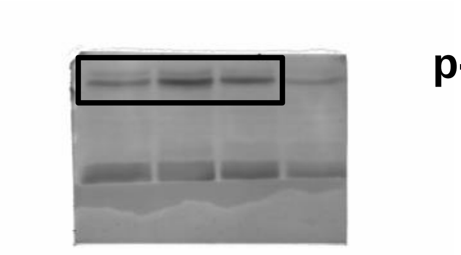

p-c-Jun 50 kDa

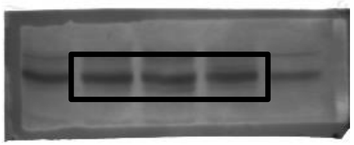

c-Jun 39 kDa

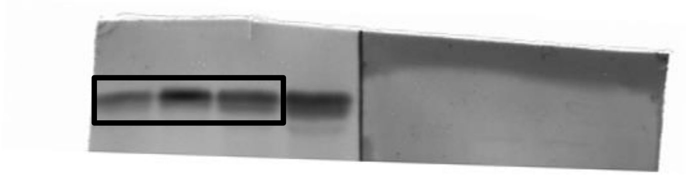

p-ERK1 44 kDa

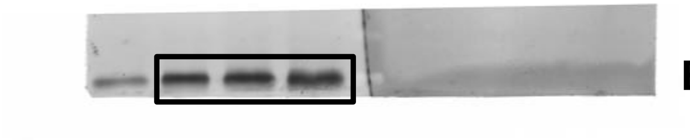

ERK1 44 kDa
